# Differential Impact of Social Distancing on COVID-19 Spread in the U.S.: By Rurality and Social Vulnerability

**DOI:** 10.21203/rs.3.rs-798357/v1

**Published:** 2021-09-14

**Authors:** Asal Pilehvari, Wen You, Jiangzhuo Chen, John Krulick, Srini Venkatramanan, Achla Marathe

**Affiliations:** University of Virginia; University of Virginia; Biocomplexity Institute, University of Virginia; Biocomplexity Institute, University of Virginia; Biocomplexity Institute, University of Virginia; Biocomplexity Institute, University of Virginia

**Keywords:** Social distancing, COVID-19 Pandemic, Spatial dependence, Spatial Durbin Model, Urban-rural disparities, Social vulnerability, Epidemiology Curve flattening, I18, H7, C31, C33

## Abstract

**Background.:**

To quantify lessons learned to better prepare for similar pandemic crisis in the future, we assess the overall impact of social distancing on the daily growth rate of COVID-19 infections in the U.S. during the initial phase of the pandemic and the impacts’ heterogeneity by urbanity and social vulnerability of the counties. The initial phase is chosen to purposely identify the essential and largest impact of the first-line of defense measure for similar pandemic: social distancing.

**Methods.:**

Spatial Durbin models with county fixed effects were used to account for spatial dependencies and identify spatial spillover effects and spatial heterogeneity.

**Results.:**

Besides the substantial curve flattening effects of social distancing, our results show significant spillover effects induced by neighboring counties’ social distancing levels even in the absence of significant within-county effects. Urban and areas with high social vulnerability are the ones benefit the most from social distancing and high level of compliance is needed. Moderate level is enough in reaching the peak marginal impact in rural and areas with low social vulnerability.

## 1 Background

By the end of July 2020 (the first phase of the pandemic in U.S.), COVID-19 had infected more than 4.6 million individuals and caused more than 155,000 deaths in the U.S. Given the novelty of the virus, and the absence of vaccines and pharmacological solutions, social distancing is the biggest control mechanism during the initial phase of this type of pandemic. The fast spreading speed of COVID-19 brings with it sever health, economic and sociopolitical consequences (Baker et al., 2020; Cajner et al., 2020; Coibion et al., 2020a, 2020b; Dunn et al., 2021). Furthermore, it has disproportionately hit the low income, minority and vulnerable populations. Low-income sub-populations have exhibited lower levels of compliance to social distancing due to occupation specific inflexibility (Drago and Miller, 2010; Lou et al., 2020; Wright et al., 2020). Stay-at-home orders did not significantly alter work trip patterns for essential businesses such as grocery stores, garbage collection, postal services, construction work etc. which account for majority of the low-income populations’ employment (Drago and Miller, 2010; Lou et al., 2020). The lack of compliance to social distancing along with health care access inequalities result in widened infection risk gaps across socio-economic and racial-ethnic subgroups (Bruce, 2008; Kendall et al., 2020; Leclere et al., 1994). For example, recent research showed that counties with higher minority population and lower socioeconomic status experienced significantly higher COVID-19 death and infection rates (Nayak et al., 2020). As efficacious vaccines are invented and their supply chain log roadblocks are resolved, we are seeing the light at the end of the tunnel. Now more than ever is the time for us to gather lessons learned in order to be better prepared for the future ones.

However, it has been recognized that it is difficult to establish causal inference in this context due to the presence of spatial and temporal confounders which influence both the speed of the spread of infection and the levels of compliance to social distancing (Courtemanche et al., 2020). Examples of such confounders are: state/county specific prevention measures such as requirement for wearing masks, restrictions on international and domestic travel, testing, contact tracing, and local norms and perceptions towards social distancing etc. Furthermore, those state/county level requirements and measures are rapidly changing over time. There is a significant heterogeneity in levels of compliance to social distancing directives throughout the U.S. and the distribution is shown to be correlated with local norms, perceptions, social-economic profiles and political leanings (Bodas and Peleg, 2020; Lou et al., 2020; Wright et al., 2020).

A lot of these confounders are either unobserved or difficult to measure directly. In this study we examine the impact of social distancing on the growth rate of daily confirmed COVID-19 cases at county level. To address the challenges of directly measuring and controlling confounders, we implement Spatial Durbin Models with county fixed-effects to indirectly control for time-invariant and county-specific unobservables while accounting for spatial dependence between counties. The spatial econometric model indirectly controls for spatial confounders through recognizing the spatial contiguity of counties and through county level fixed effects. The models acknowledge that infectious diseases, such as COVID-19, follow spatial patterns i.e., the infection rates are correlated with geographic proximity and connectivity through mobility among communities (Langford, 2002).

Our analysis focuses on the time period corresponding to the first wave of COVID-19 U.S. pandemic that goes from mid-March till mid-June, which corresponds to the highest levels of social distancing compliance (Barry et al., 2021). We chose this time period based on the fact that procedures such as social distancing are the first line of defense in the early phase and quantitative evidence is needed for current and future infectious disease public health prevention and intervention.

Our overall goals are to: 1) quantify the direct and indirect marginal effects of social distancing compliance in reducing the growth rate in infections; 2) assess the disparity in marginal effects between rural and urban areas, and between areas with high or low social vulnerability. Specifically, the second goal aims at quantifying the differential impacts of social distancing which is of high policy relevance. It is motivated by the fact that region-specific factors such as healthcare access, population density, and racial/ethnicity composition can be quite different across urban and rural areas. For instance, rural areas are characterized by sparse populations and lower housing density compared to urban areas. Therefore it is not surprising that regional patterns of disease spread and social distancing compliance will be different. Similarly, those differences are expected to be observed in low versus high vulnerability areas, as characterized by the social vulnerability index (SVI) provided by the CDC (U.S. Centers for Disease Control and Prevention). The SVI for each U.S. county considers community level poverty, socio-economic status, transportation access, disabilities and housing composition, among other variables. It provides another way to examine potential subgroup differences of social distancing on the spread of COVID-19 beyond traditional dimension of mere population size. Our second goal of quantifying impact heterogeneity across areas may provide evidence for designing area-based customized policies for achieving cost-effectiveness.

**Figure 1:**
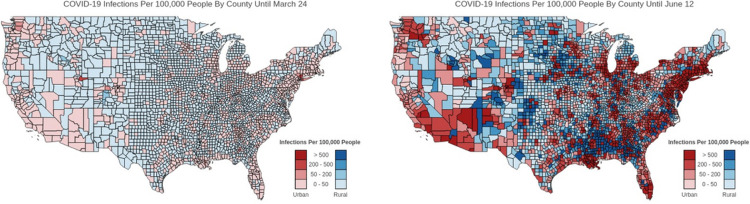
Spatial spread of cumulative infections per 100,000 people by urban and rural counties in the U.S. SOURCE Authors’ analysis of data from the University of Maryland COVID-19 Impact Analysis Platform.

**Figure 2:**
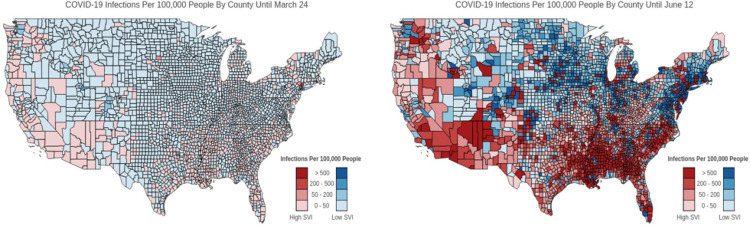
Spatial spread of cumulative infections per 100,000 people by counties with high and low social vulnerability index (SVI) in the U.S. SOURCE: Authors’ analysis of data from the University of Maryland COVID-19 Impact Analysis Platform. NOTE: Spatial spread of cumulative infections per 100,000 people by counties with high and low social vulnerability index (SVI) in the US. High SVI counties are the ones in the top half of SVI distribution and Low SVI counties refer to those in the bottom 50% of the SVI distribution.

Figure 1 shows cumulative infections at the beginning (left) and at the end of the study period (right) for urban vs. rural counties. Figure 2 shows cumulative infections at the beginning (left) and at the end of the study period (right) for high vs. low SVI counties. As can be seen in these plots, the infected areas are highly spatially clustered, meaning adjacent counties experience similar levels of infections. Therefore, conventional regression models (e.g., Ordinary Least Squares) that impose independence assumption on observations and ignore the spatial dependence among them will produce biased and inconsistent inferences (Wooldridge, 2016). Another reason is that spatially adjacent counties are more likely to share similar norms and perceptions which can include acceptance towards social distancing. Therefore, in the context of infectious disease such as COVID19 that is spread primarily via close contact, it is important to quantify and disentangle direct effects (within county and feedback effects) and indirect effects (across counties spillover effects) for understanding the value of coordinated efforts among neighboring counties.

A few studies have investigated the impact of various social distancing-based interventions on the spread of COVID-19 infections. In particular, a study finds that shelter-in-place order along with closure of bars, restaurants and entertainment outlets, on average reduced daily growth rate of confirmed COVID-19 cases by 5.4% after 1–5 days, 6.8% after 6–10 days, 8.2% after 11–15 days, and 9.1% after 16–20 days across U.S. counties (Courtemanche et al., 2020). However, there are no studies in the literature that have quantified the direct and indirect effect of social distancing and examined the differential impact of social distancing on COVID-19 infections in rural vs. urban, and low vs. high SVI counties in the U.S.

Our findings support the importance of social distancing in slowing down infections during the early phase of the pandemic. Notably, the spatial estimation uncovers significant spillover effects on infection rate reduction in a typical county (i.e., rate reduction caused by social distancing compliance in its neighboring counties) and provides support for multi-county social distancing coordination efforts. Our results further reveal that those counties with high social distancing index (SDI) (i.e., those with SDI level higher than the median of the SDI distribution) on average experienced about 1.84% lower daily infection growth rates compared to those counties with low SDI. This difference is larger when comparing urban areas to rural ones (i.e., 1.88% rate differences), and even larger when comparing areas with high vs. low social vulnerability index (i.e., 2.00% rate differences). Moderate level of social distancing is found to be most effective (i.e., generating the largest rate differences as compared to those non-compliance counties) in rural and low SVI areas and contributes to reduction in daily infection growth rate by 1.5% and 1.2%, respectively. Results of this paper highlight the importance of collateral planning and coordinating with the geographically adjacent counties in flattening the epidemic curve.

## 2 Data

We use data from the “COVID-19 Impact Analysis Platform” made publicly available by the University of Maryland (UMD)(MTI, n.d.; Zhang et al., 2020). This database provides information on COVID-19 related infections, deaths, social and economic indicators, social mobility, as well as testing and tracing information at a county level for the entire U.S. on a daily basis. Specifically, it includes daily number of newly confirmed COVID-19 infections, cumulative death rate, number of active cases etc. It also provides several mobility and social distancing measures such as population movement within and out of county, percentage of residents staying at home, average number of all trips per person per day, daily average person-miles traveled, daily percentage of all trips that cross county borders, percentage of all trips that cross state borders per day, and daily social distancing index.

Furthermore, the UMD data set also contains the Social Distancing Index (SDI) developed by the Maryland Transportation Institute (MTI) using a series of county-level mobility measurements and changes compared to pre-COVID-19 levels, with highest weight given to stay-at-home levels.The SDI is provided at daily frequency and therefore each data point represents the degree of mobility reduction from pre-COVID benchmark for a specific county on that day: i.e., a lower chance for a close person-to-person interaction. It is an integer between 0 (no social distancing at all) and 100 (100% of the residents in the county follow social distancing). It is also important to control for the counties’ virus exposure level and testing capacity so that the social distancing effect is compared among counties with similar exposure and prevention efforts. Therefore, we control in our models the following variables: number of COVID-19 tests done per 1000 people (with 2 days lag) and number of residents already exposed to COVID-19 (with 14 days lag).

The outcome variable of interest is the daily growth rate of confirmed COVID-19 cases. If a county had zero confirmed COVID-19 cases, we consider its infection growth rate as zero. However, since explanatory variables are entered into the model with 14-day lag, our daily growth rate of COVID-19 cases starts from late March when only 2% of the counties had zero confirmed COVID-19 cases. Even though the death count is relatively more accurate than the case incidence rate, we focus on studying the impact of the social distancing on the growth rate of infections since the social distancing measures are meant to mainly slow down the spread of the virus and not directly affect the death rate. Although the number of cases correlates with the number of deaths, the death rate depends on a variety of other factors as well such as age, comorbidities, access to health care, availability of hospital beds, access to health insurance and ventilators etc. (Guan et al., 2020)

The period of analysis starts on March 19 (i.e., the start of the lock down period), and ends on June 12, 2020 (end of first wave). In order to avoid the linear and constant marginal impact assumption when using the SDI as a continuous variable, we convert the SDI into discrete categories. It is more informative to quantify the impact of SDI in a relative sense between groups since there is no evidence to support the exact ‘optimal’ level of SDI. For example, a binary version of SDI would measure the extent to which more compliant counties do better in terms of slowing down the infection rates compared to counties that are less compliant. Based on these reasons, we examine two relative measures of SDI. First, we use a dummy variable that indicates whether or not the county’s SDI level is greater than the median of the SDI distribution of all U.S. counties. To facilitate readability, counties located in the upper 50% of SDI distribution are called “compliant counties” and in the bottom half of the distribution are called “non-compliant counties”. To further explore nonlinear impact of SDI and facilitate policy-making, we group counties by SDI quartiles and represent those quartile indicators in the empirical models using dummies. We call counties in the first/lower quartile “non-compliant counties”, in the second quartile “low-compliance counties”, in the third quartile “moderate-compliance counties” and in the fourth/upper quartile “high-compliance counties”.

To conduct subgroup analysis, we first classify counties as rural or urban using the Rural Urban Continuum Codes (2013) developed by the Economic Research Services of the Department of Agriculture and the Rural Health Research Center at the University of Washington. Counties with code values of 1 to 5 are classified as urban and those with code values of 6 to 9 are considered as rural. In a total of 3,142 counties across U.S., 1,472 counties are classified as urban and 1,668 as rural.

The other subgroup analysis is based on the Social Vulnerability Index (SVI). The CDC has developed the SVI for U.S. counties to capture social determinants of health-based inequalities which in turn impact communities’ ability to deal with health crisis (Marmot et al., 2008). A high value of SVI implies a high level of vulnerability in the county. It is based on 15 social factors that measure four dimensions of overall vulnerability: socioeconomic status, household composition and disability, minority status and language, housing type, and transportation. Needless to say that levels of social distancing varies with different social vulnerability status. Therefore, it is important to understand the differential impact SDI has across counties with different SVI status. We use the 2018 SVI data to group counties into high (i.e., those with the SVI level above the median of the SVI distribution) and low (i.e., those with the SVI in the bottom half of the SVI distribution) socially vulnerable counties.

Table 1 provides data description and summary statistics of the variables and the comparison between rural vs. urban and low vs. high SVI counties. On average, daily infection growth rate is 5.33% with a maximum of 2833%. During the time period of analysis, the average social distancing index level is 33.52. The average percentage of daily trips across state borders is 5.96%, and the mean daily number of residents exposed to COVID-19 is 5.33. On average 23.05 COVID-19 tests were done per 1000 people across all counties. All variables exhibit statistically significant differences between rural and urban, and between low and high SVI counties. On average, rural areas see lower daily infection growth rates compared to urban areas while rural areas exhibit relatively lower SDI level as compared to urban areas. Residents in rural areas had more out of state trips than residents in urban areas. As expected, high SVI counties have larger daily infections growth rate (i.e., 5.83 vs 4.83) and lower levels of SDI as compared to low SVI ones (i.e., 32.28 vs 34.76). These disparities support our subgroup-based approach to quantify the SDI impact heterogeneity.

Our empirical analysis accounts for potential observed confounders including daily number of COVID-19 tests (per 1,000 people), number of point of interests for crowd gathering in counties (per 1,000 people), number of residents exposed to COVID-19 (per 1,000 people), and percentage of daily trips across state borders. Due to 14-day incubation period of COVID-19, these variables as well as social distancing compliance enter into our empirical models with 14-day lag, except for number of testing which is used with a 2-day lag. On average, it took about two days to get COVID-19 test results. The county fixed-effect controls all county-level time-invariant characteristics such as racial and ethnicity profiles, socioeconomic levels, etc.

## 3 Methods

Spatial econometrics models are gaining popularity in the investigation of disease outbreak especially those mainly transmitted via in-person contacts (Brockmann, 2010). It not only provides consistent estimates of the intervention effects by accounting for spatial dependency but also has the ability to disentangle direct (which includes feedback effects) and indirect effects. Specifically, the method enables us to calculate a total direct effect of county A’s social distancing on daily infection growth rate that includes the feedback/boomerang effect caused by spatial dependency (i.e., the part of its infection growth rate reduction reflected back from its neighboring counties’ reduction in infection rate which in turn was caused by county A’s compliance). Furthermore, we can calculate the indirect effect (or spillover effect) which is the impact of county A’s neighboring counties’ social distancing compliance on county A’s daily growth rate of infections.

Specifically, we estimate Spatial Durbin Models with county fixed-effects to extract the spatial dependencies in daily growth rate of infections in U.S. counties during the initial phase of COVID-19 spread (LeSage and Pace, 2009). Our model includes county fixed effects but not time fixed effect to allow more efficiency. Furthermore, the daily growth rate calculation has already taking time trend into consideration. Time fixed effect is not included specifically because the surge in infections follows spatial pattern rather than temporal: i.e., the rise and plateau in Seattle and west coast are not synchronized with east coast temporally. The model specification is defined as follows:

$${\text{y}}_{\text{nt}}=\rho {\text{Wy}}_{\text{nt}}+\alpha {\text{l}}_{n}+{\text{X}}_{\text{nt}-14}\beta +{\text{WX}}_{\text{nt}-14}\theta +\mu_{\text{n}}+\varepsilon_{\text{nt}}$$

Where, y_nt_ = (y_1t_, y_2t_, y_3t_, …, y_nt_)′ is a n × 1 vector of county-specific daily growth rate of COVID-19 infections in period t. W is the spatial weight matrix and Wy_nt_ is the spatial lag, ρ reflects the strength of spatial dependence between counties, and l_n_ denotes n × 1 vector of ones. X_nt−14_ contains time varying exogenous explanatory variables, i.e. social distancing and other county specifics with 14 days lag to impose temporal exogeneity and avoid the simultaneity problem of social distancing compliance with COVID-19 Cases,^11^ θ is a k × 1 vector of exogenous spatially lagged explanatory variables. Unobserved county-fixed effects are denoted as μ and ε_nt_ represents the i.i.d error term.

To capture spatial linkages between counties i and j, we use contiguity matrix that contains values of either 0 or 1, where 1 refers to the case that county i and j are adjacent (i.e., share geographic borders) and 0 otherwise.^12^ The spatial weight matrix W is a row-statistic conversion of contiguity matrix of 0 and 1 that captures spatial linkages between counties i and j (with i, j = 1, …, n). The contiguity matrix contains values of either 0 or 1, where 1 refers to the case that county I and j are adjacent (i.e., share geographic borders) and 0 otherwise. W is positive definite with zero diagonal.^13^ The contiguity matrix is obtained using geographical coordinates of counties available in Census 2018-shape files.^14^

We use Maximum Likelihood Estimation method to estimate parameters in spatial regression. The null hypothesis of cross-sectional independence is rejected^15^ in the full sample and all sub-samples which provide statistical support for spatial specification and its importance for consistent inference. To begin, we first report estimation results based on naive pooled Ordinary Least Squares (OLS) regression which ignores spatial dependency and then compare with the spatially dependent analysis. In all empirical analysis, we present robust standard errors that recognize that the daily data is clustered within county level.

## 4 Results

### 4.1 No Spatial Dependence Assumed Between Counties

**Figure 3:**
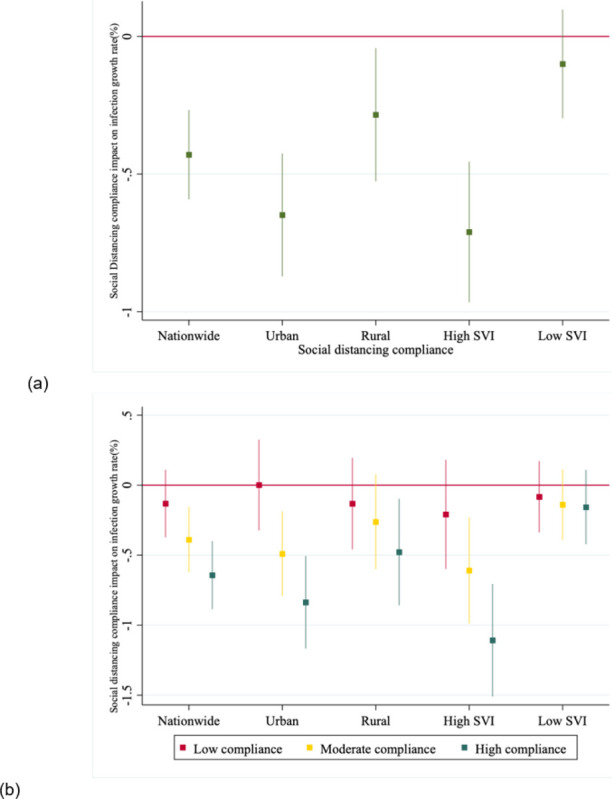
OLS estimated impact of social distancing compliance on the daily growth rate of COVID-19 infections in the U.S. (a) OLS estimated impact of different levels of social distancing compliance on the daily growth rate of COVID-19 infections in the U.S. (b) OLS estimated impact of different levels of social distancing compliance on the daily growth rate of COVID-19 infections as compared to non-compliant counties in the US. NOTES Figure (a) shows the impact with SDI measured in binary values and Figure (b) shows the impact with SDI measured in quartiles. Counties in the bottom 25% of SDI distribution are called non-compliant counties which is the baseline in the estimations, those in the second quartile are low-compliance, third quartile are moderate-compliance, and the fourth quartile are the high-compliance counties. High SVI counties refer to those located in the top 50% of the SVI distribution and low SVI indicates counties in the bottom half of the SVI distribution. The time period of study is March 19 to June 12, 2020. In all estimations, standard errors are heteroskedasticity-robust and clustered by counties. Bars present 95% confidence intervals.

Figure 3a presents parameter estimates of the impact of social distancing on daily growth rate of infections at a county level, using naive pooled OLS regression which ignores spatial dependence between counties. The results show that counties with SDI above median levels, on average experience 0.43% lower daily infection growth rate as compared to counties with SDI levels in the bottom half of the SDI distribution. The sub-sample analysis shows that, compared to rural counties, urban areas on average experience a larger infection growth rate gap between high SDI and low SDI counties (about 0.65% in urban and 0.28% in rural). The more compliant counties in socially vulnerable areas (i.e., high SVI areas) experience larger reduction in infection growth rate (about 0.71%) whereas SDI compliance does not have statistically significant impact on low SVI areas.

We further refine the SDI compliance groups by SDI distribution quartiles. Results are presented in Fig. 3b for four groups of counties with different relative SDI levels (the base comparison group contain counties with SDI in the bottom quartile of the distribution (“non-compliant” ones)). It shows that in order to have a statistically significant effect on flattening the curve, counties need to be at least moderately compliant to social distancing (i.e., SDI level should be in or above third quartile of the distribution).Similar to what was observed in Fig. 3a where SDI took a binary value, urban areas have a larger reduction in infection rate from SDI compliance as compared to rural areas, and it has the largest impact in socially vulnerable areas (1.11% daily infection growth rate gap between high-compliance counties and non-compliant counties). However, this naive regression model does not control for spatial dependence between counties and cannot disentangle the direct and indirect effects which are of public health policy importance.

### 4.2 With Spatial Dependence Considered

**Figure 4:**
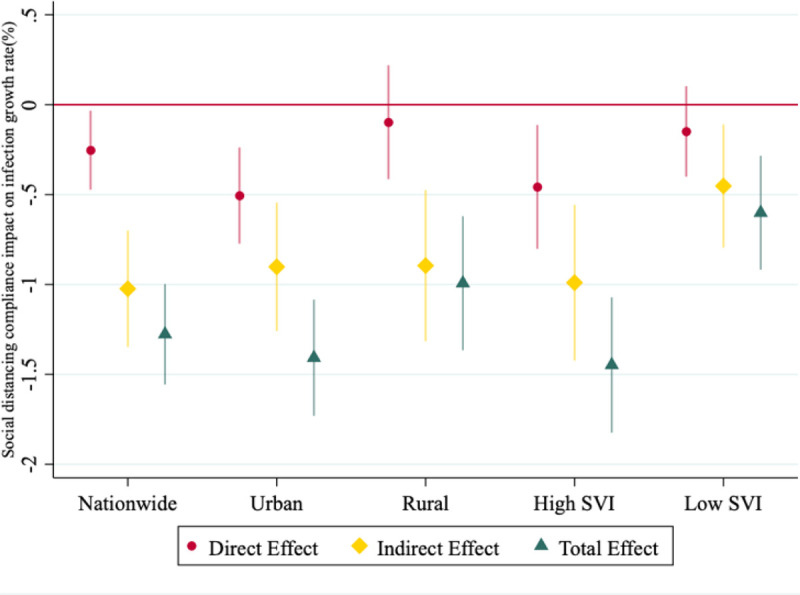
Spatial estimation of social distancing compliance impact on daily growth rate of COVID-19 infections across U.S. counties between March 19 to June 12, 2020 NOTES The figure shows direct, indirect and total effect of social distancing on daily growth rate of COVID19 infections, for the binary SDI case. High SVI counties refer to those located in the top 50% of the SVI distribution and low SVI indicates counties in the bottom half of the SVI distribution. Direct effect refers to within county effect and Indirect effect indicates the effect of neighboring counties on a county’s infection growth rate. The total effect is the sum of direct and indirect effect. Standard errors are county cluster robust. Bars present 95% confidence intervals.

Figure 4 presents results of empirical estimation based on Spatial Durbin Model for the U.S., urban, rural, high SVI and low SVI regions. The direct effect shows social distancing in a county significantly reduces rate of infections within the same county. In particular, on average, social distancing compliant counties experience about 0.25% lower infection growth rate compared to non-compliant ones in the national sample.

More importantly, the indirect effect of social distancing compliance is much larger: i.e., neighboring counties’ SDI, all together, will result in about 1.02% reduction in a typical county’s daily infection growth rate. This indirect effect is more than four times the direct effect. The much larger indirect effect is no surprising since the indirect effect is the sum of all the neighboring counties’ effect. This evidence will be masked in models that do not account for spatial dependency. The both statistically significant and public health impact relevant indirect effect of SDI compliance highlights the importance of coordinated planning among counties that share common geographic boarders to combat the spread of COVID-19. The total effect of social distancing combines the direct effect and indirect spillover effect and shows a difference of 1.28% in daily infection growth rate between compliant and non-compliant counties.

This pattern of relatively larger indirect effects persists in sub-sample analysis of urban, rural and socially vulnerable counties. Most direct and indirect effects are all statistically significant with the only exception that the direct effects are not statistically significant in rural areas and areas with low SVI. Even though rural and low SVI areas do not see statistically significant impact of their own SDI compliance on slowing down the infections (i.e., null direct effects), there are significant indirect effects on flattening the epidemic curve as a result of neighboring counties’ efforts. Furthermore, the magnitudes of the indirect effects are similar in urban, rural and socially vulnerable areas signaling the importance of coordinated efforts in those areas.

Socially vulnerable areas see the largest total effect of social distancing compliance (about 1.45% growth rate differences between high and low compliance counties) followed by urban areas (about 1.41% growth rate differences between high and low compliance counties). In case of the OLS regression which ignores the spatial dependency between neighboring counties, we do not find statistical evidence to support the effectiveness of social distancing compliance in low SVI areas. However, the SDM model which accounts for spatial dependency finds statistically significant indirect effects of social distancing on infection control and flattening of the curve.

**Figure 5:**
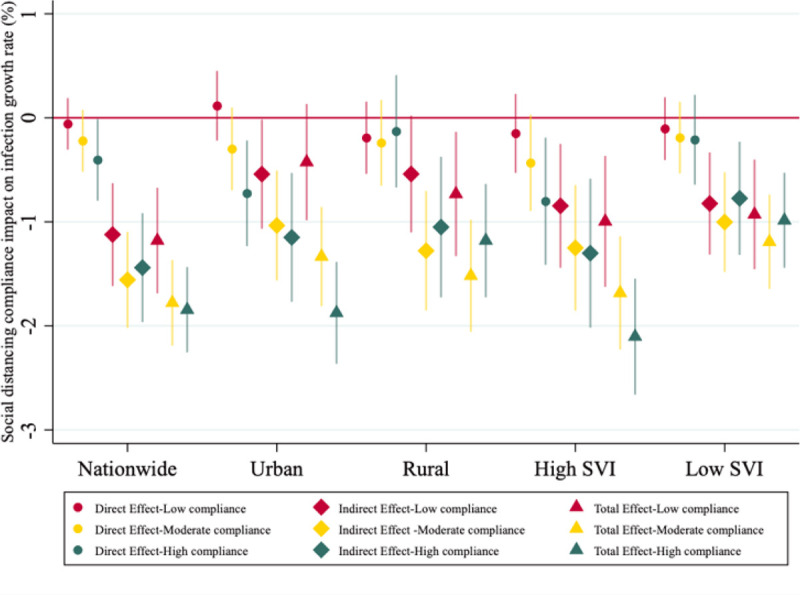
Impact of various social distancing compliance levels on COVID-19 infection rate assuming spatial dependence between neighboring counties NOTES Direct effect refers to within county effect and the Indirect effect refers to neighboring counties’ effect on growth rate of infection. The total effect is the sum of direct and indirect effect. Counties in the bottom 25% of SDI distribution are called non-compliant counties which is the baseline in the estimations, those in the second quartile are low-compliance, third quartile are moderate-compliance, and the fourth quartile are the high-compliance counties. High SVI counties refer to the ones in the top 50% of the SVI distribution and low SVI in the bottom half of the SVI distribution. Standard errors are county cluster-robust. Bars present 95% confidence intervals.

To further investigate potentially heterogeneous effect of social distancing, we estimate the spatial models with three group dummies that categorize counties according to their SDI quartiles and present the results in Fig. 5. Results show that high-compliance counties (i.e., those with SDI in the fourth quartile of the distribution) are the ones experiencing statistically significant direct effect on reduction of infection growth rates as compared to non-compliant counties (i.e., those with SDI in the first quartile of the distribution): about 0.4% lower infection growth rate as a result of their own compliance to social distancing, compared to non-compliant counties. This pattern also holds in urban areas (about 0.73% lower infection growth rate in high compliance counties compared to non-compliant ones). Similar to the binary SDI model, rural and low SVI areas do not see statistically significant direct effect of social distancing. Noticeably, moderate and high-compliance counties show significant direct effect on infection control in socially vulnerable areas i.e., compared to non-compliance counties, there are an average of −0.43% in moderate-compliance counties and − 0.80% in high-compliance counties.

Similar to the binary SDI model, all indirect spillover effects induced from social distancing compliance in neighboring counties are significant and show much larger public health impact significance (i.e., larger magnitudes). Noticeably, moderate-compliance level has the largest indirect effects and total effects on infection control in the national sample and in rural and socially less vulnerable areas, whereas high-compliance level is needed to achieve the largest effects in urban and socially vulnerable areas. Possible explanations for this result could be the fact that rural and low SVI areas have lower population density, sparse housing and more open spaces that social distancing compliance effect can be achieved without having to abide to exact measures. For example, stay-at-home may not be needed for rural areas where the population density and housing density are low to achieve crowd avoiding and physical distancing goals as compared to urban areas which have high population density and more mobile.

In socially vulnerable areas, complex interaction of socioeconomic factors (such as wage jobs, low income, inflexible work conditions, etc.) contribute to the need for higher levels of social distancing to curb the spread of COVID-19 infection. For example, people with low socioeconomic profiles are often working for essential businesses which are exempt from lock downs; or they are working in jobs that require human contact such as child-care, nursing, house-cleaning, cooking etc. which limits their ability to fully comply with social distancing regulations and results in greater exposure to risk of infection.

Both Fig. 4 and Fig. 5 show that the direct effect of social distancing within counties may be minimal and/or null but it produces meaningful indirect effects at lowering COVID-19 infection growth rate due to spatial dependence of counties. Figure 5 further confirms the importance of compliance to social distancing. Based on the total effect results, counties with moderate social distancing compliance levels show a sizable reduction in infection growth rates. Even counties that have low compliance, there is a sizable and significant reduction nationwide and in sub-samples, except for urban areas.

Considering the daily fluctuation in the growth rate of infection, we also examine the impact of social distancing compliance on a 3-day moving average of infection growth rates and our results remain unchanged except a slight reduction in magnitudes.

## 5 Discussion

In this paper, we investigate the effectiveness of social distancing and its heterogeneity in combating the COVID-19 pandemic. Using spatial econometric methods, we find significant reduction in COVID-19 infection growth rate as a result of social distancing in the U.S. counties, especially among urban counties and counties with high SVI. Our results demonstrate significant and sizable spillover effects induced by social distancing in neighboring counties for nationwide samples and all sub-samples, even when within county effects are absent. Within county social distancing significantly lowers the growth rate of infections except in rural and low SVI areas. Our findings provide empirical evidence of spatial dependence between counties and highlight the need to coordinate planning and mitigation efforts with the geographically adjacent counties to successfully combat COVID-19 pandemic.

Policies that enforce social distancing, such as stay-at-home, are economically costly (Birge et al., 2020), and therefore we need to identify areas which are likely to benefit most from such policies. Overall, the effect of social distancing has been found to be higher in urban than in rural areas. Results indicate highest levels of social distancing compliance is needed in urban areas, whereas moderate levels of social distancing compliance will suffice in controlling the spread in rural areas, probably because of the low population and housing density in those counties. Similarly, highest levels of social distancing are needed in flattening the epidemic curve in high SVI areas but moderate levels may be sufficient in low SVI areas. The examination of areas with high and low SVI adds socio-economic and disaster readiness dimensions to the mainly population size categorization done by rural vs. urban analysis.

Those sub-group analysis will guide policy makers and public health officials in setting up appropriate levels of social distancing goals and collaborated efforts such as community-based volunteer networks (Kobokovich et al., 2020), at least until a medical solution is available to control the pandemic. It also highlights the importance of having clear and coherent national and local plans that can be implemented in a cost-effective fashion across the country. A patchwork of policies that are applied piece meal by counties and states who independently decide what is best for them, has not been working and will not work as shown by this analysis. There are a lot of spatial dependencies between regions that should be considered for a cost-effective public health response.

## Conclusions

In summary, our findings contribute in three aspects to the literature: 1) our methods confirm those found in the literature that social distancing efforts have statistically significant impacts on slowing down the spread of the virus and the effects are of nontrivial size; 2) our methods further reveal that the social distancing impacts are heterogeneous across geographic dimensions; and 3) the geographically-targeted orders and local county level shared-decision making will likely achieve higher benefits in terms of virus control and bring much smaller economic interruptions.

There are a number of limitations to this analysis. First, it is well-known that number of confirmed COVID-19 cases may not represent the actual cases of COVID-19 infections due to lack of testing capacity, asymptomatic infections, access to healthcare etc. (Stock et al., 2020). For instance, it is highly likely that people with mild symptoms did not seek medical care and hence did not get counted among the confirmed cases. However, our paper findings on the marginal impact of SDI and the disparity of the impact across rural/urban and low/high vulnerability areas will still be valid and robust as long as the under-reporting is uniformly distributed. Second, the social distancing index may not precisely capture the level of social distancing in a society. The SDI metric is based on mobility information obtained through mobile devices that need users to enable their devices for data collection (e.g., GPS history). This type of data collection by nature will exclude people who do not have smart phones, or do not have their devices enabled for data collection. To the best of our knowledge, UMD social distancing index not only includes the most comprehensive and relevant dimensions especially those capturing spatial dependencies in mobility (e.g., percentage of out-of-county trips) but also covers all 3,142 counties across the U.S. The other available social distancing related datasets such as the Google community mobility data and Apple mobility data are either proprietary, or not available at all county levels (e.g., Google community mobility data covers only 2,795 counties in the U.S.

## Figures and Tables

**Table 1:** Data description and Summary Statistics for pooled daily data (March 19 - June 12, 2020)

	Nationwide			Rural	Urban	Urban-Rural Disparity	Low SVI^a^	High SVI^b^	Low SVI-High SVI disparity
	Mean	Min	Max	Mean	Mean		Mean	Mean	
Infection growth rate (%)	5.33 (0.22)	0	2833	4.03 (0.21)	6.80 (0.23)	−2.77*** (0.00)	4.83 (0.20)	5.83 (0.24)	−1*** (0.00)
Social distancing index	33.52 (15.14)	0	100	30.31 (14.18)	37.15 (15.38)	−6.835*** (0.06)	34.76 (15.56)	32.28 (14.61)	2.481*** (0.06)
Out of state trips (%)^d^	5.96 (8.13)	0	100	6.592 (8.63)	5.247 (7.48)	1.345*** (0.03)	6.298 (8.26)	5.624 (7.99)	0.675*** (0.03)
COVID-19 Exposure per 1000 people^e^	5.33 (6.46)	0.01	44.32	4.634 (5.04)	6.123 (7.68)	−1.488*** (0.02)	5.731 (6.86)	4.932 (6.01)	0.799*** (0.02)
Number of Tests done per 1000 people^f^	23.05 (22.00)	0.02	181.1	22.55 (21.33)	23.6 (22.73)	−1.046 (0.08)	23.25 (22.40)	22.83 (21.53)	0.418*** (0.08)
People older than 60(%)	25.27 (5.65)	6	65.00	27.28 (5.49)	23.02 (4.90)	4.257*** (0.02)	26.31 (6.07)	24.23 (4.98)	2.078*** (0.02)
African-Amerlicans (%)	8.92 (14.46)	0	.87.40	7.70 (15.18)	10.32 (13.48)	−2.611*** (0.06)	3.37 (5–81)	14.47 (17–97)	−11.10*** (0.05)
Hispanic-Americans (%)	9.26 (13.79)	0	99.10	8.52 (14.16)	10.08 (13.22)	−1.604*** (0.05)	5,835 (7.43)	12.6 (17.32)	−6.714*** (0.05)
Median income	51,580.4 (13700)	20,188	136,268	46,686 (10414.80)	57,161 (14800.90)	−10465.5*** (48.83)	58.650. (13630.10)	44,520 (9479.70)	14130.9*** (45.17)
Male	50.09 (2.38)	41.39	79.00	50.54 (2.78)	49.57 (1.68)	0.971*** (0.01)	50.07 (177)	50.1 (2.86)	−0.0310*** (0.01)
Number of points of interests	131.56 (42.33)	8	699.00	137.70 (48.73)	124.70 (32.05)	13.05*** (0.16)	147.2 (46.23)	116 (30.94)	31.21*** (0.15)
Observations	270212			270040			270126		

NOTES: This Table reports summary statistics of selected variables of 3142 counties using COVID-19 Impact Analysis Platform database available from the University of Maryland for the time period between March 19 to June 12. Nationwide sample size (county-by-day observations; 3,142 counties for 86 days) is 270,212. Two counties are excluded in the rural-urban sample due to missing classifications. Therefore, 1,472 counties are classified as urban and 1,668 are as rural, which makes a total of 270,040 counties in the urban-rural sub-sample. Social vulnerability index (SVI) was also missing for a county which is excluded in the SVI sub-sample analysis. Therefore, 3,141 counties are classified in two sub-samples of High and Low SVI based on the median of SVI 2018 distribution. Low SVI: counties in the bottom half of the SVI distribution High SVI: counties in the top 50% of the SVI distribution.

c The Social Distancing Index (SDI) is defined by Maryland Transportation Institute (MTI) as follows: SDI =0.8*[% stay-at-home + 0.01* (100 - %stay-at-home) * (0.1*% reduction all trips + 0.2*% reduction work trips + 0.4*% reduction non-work trips + 0.3*% reduction travel distance)] + 0.2*% reduction out-of-county trips. It is an integer between 0 (no social distancing at all) and 100 (100% of the residents in the county follow social distancing)

d Percentage of all trips that cross state calculated by MTI.

e It is calculated as the number of residents already exposed to COVID-19 per 1000 people.

f Number of points of interests for crowd gathering per 1000 people calculated by MTI. Asterisks show that the difference between the urban vs. rural and high vs. low SVI is statistically significant i.e.,

* p<0.10,

** p<0.05,

*** p<0.01.

## Data Availability

The datasets used and/or analysed during the current study are available from the corresponding author on reasonable request.
